# Binocular balance across spatial frequency in anisomyopia

**DOI:** 10.3389/fnins.2024.1349436

**Published:** 2024-01-25

**Authors:** Nan Jiang, Yang Zheng, Mengting Chen, Jiawei Zhou, Seung Hyun Min

**Affiliations:** School of Ophthalmology and Optometry, Affiliated Eye Hospital, State Key Laboratory of Ophthalmology, Optometry and Vision Science, Wenzhou Medical University, Wenzhou, China

**Keywords:** anisomyopia, binocular vision, contact lenses, axial length, visual acuity

## Abstract

**Purpose:**

Anisomyopia is prevalent in myopia and studies have reported it exhibits impaired binocular function. We investigated the binocular balance across spatial frequency in adults with anisomyopia and compared it to in individuals with less differences in refractive error, and examined whether ocular characteristics can predict binocular balance in anisomyopia.

**Methods:**

Fifteen anisomyopes, 15 isomyopes and 12 emmetropes were recruited. Binocular balance was quantitatively measured at 0.5, 1, 2 and 4 c/d. The first two groups of the observers were tested with and without optical correction with contact lenses. Emmetropes were tested without optical correction.

**Results:**

Binocular balance across spatial frequency in optically corrected anisomyopes and isomyopes, as well as emmetropes were found to be similar. Their binocular balance nevertheless still got worse as a function of spatial frequency. However, before optical correction, anisomyopes but not isomyopes showed significant imbalance at higher spatial frequencies. There was a significant correlation between the dependence on spatial frequency of binocular imbalance in uncorrected anisomyopia and interocular difference in visual acuity, and between the dependence and interocular difference in spherical equivalent refraction.

**Conclusion:**

Anisomyopes had intact binocular balance following correction across spatial frequency compared to those in isomyopes and emmetropes. Their balance was weakly correlated with their refractive status after optical correction. However, their binocular balance before correction and binocular improvement following optical correction were strongly correlated with differences in ocular characteristics between eyes.

## Introduction

Binocular vision is important for maintaining depth perception (Han et al., 2018; Wang et al., 2021). Despite its importance in performing daily visual tasks, it can be imbalanced when two eyes contribute unequally (Ding et al., 2013), a phenomenon known as ocular dominance, which has physiological correlates in the primary visual cortex. For instance, when there is severe ocular dominance in favor of one eye, the response and number of neurons in the ocular dominance column from one eye can be significantly larger than those from the other eye (Sengpiel et al., 1995; Ooi and He, 2020). Studies show that binocular imbalance can exist from individuals with abnormal refractive status of one or two eyes, such as in anisometropia (Vincent et al., 2014; Zhou et al., 2016), but how it arises remains unclear.

Anisometropia is a case where two eyes, supposedly with identical gene profiles and environmental exposure throughout life, reach different refractive endpoints (Dirani et al., 2006; Ip et al., 2008). Myopic anisometropia (anisomyopia) is a unique condition of anisometropia where both eyes are myopic or one is myopic and the other is emmetropic and have a difference in spherical equivalent refraction of at least 1.00 D (Wang S. et al., 2020; Wang X. et al., 2020; Wu et al., 2021). If the difference in spherical equivalent is smaller and when both eyes are myopic, then the optical condition is referred to as isomyopia. When refractive error is absent, there is emmetropia, where little or no difference in spherical equivalent between eyes exists. Unlike others, anisomyopia has asymmetrical refractive status from both eyes. Accounting for about 30% of myopia cases, its mainly caused by the asymmetric axial lengths of both eyes (Vincent et al., 2014; Wang S. et al., 2020; Wang X. et al., 2020). Also, those with anisomyopia, even with optical correction, may have impaired binocular functions, such as fusion (Brooks et al., 1996; Jeon and Choi, 2017), stereopsis (Oguz and Oguz, 2000; Yang et al., 2013) and aniseikonia (Rabin et al., 1983; South et al., 2019). Also, the pathogeny and the mechanism underlying asymmetric refractive error development remain unclear. If ocular dominance plays a role in asymmetric eye growth as some studies suggest (Vincent et al., 2011, 2014), a clearer eye dominance in anisomyopia than in isomyopia and emmetropia should be expected. Studying anisomyopia provides an opportunity to understand whether differences in ocular characteristics between eyes can predict the degree of binocular imbalance and its extent of improvement following optical correction.

However, much remains unknown about the relationship between binocular balance and anisometropia (ex. anisomyopia). For instance, although some studies indicate that the more dominant eye is more myopic (Cheng et al., 2004; Vincent et al., 2011; Jiang et al., 2015), others support otherwise (Linke et al., 2011; Zhou et al., 2016). Also, several studies show no association between ocular dominance and refractive error (Chia et al., 2007). These discrepancies could be from using different methods to determine ocular dominance. Some methods determine motor dominance qualitatively, revealing which eye is more dominant, using the hole-in-the-card test (Ding et al., 2018). However, psychophysical methods determine sensory eye dominance quantitatively. These two types can be independent from each other (Ding et al., 2018). In most studies about eye dominance and anisometropia, qualitative methods have been used, preventing investigators from drawing a quantitative relationship between them. Moreover, because the importance of binocular balance has been increasingly recognized in the clinic (Hess et al., 2014), there is a need to clarify which clinical characteristics are associated with binocular balance in populations with a wide range of refractive error differences between eyes. This would enable researchers and clinicians to better predict the state of binocular balance and its potential improvement after treatment based on clinical characteristics of both eyes.

In this study, we measured sensory eye dominance in young adults with anisomyopia, isomyopia and emmetropia across a range of spatial frequency using a psychophysical task before and after optical correction. Contact lenses, rather than spectacles, were used for optical correction to minimize aniseikonia, which could destabilize binocular balance (Winn et al., 1988). We had two purposes in our study. First, we wanted to examine whether individuals with anisomyopia had larger binocular imbalance across spatial frequency than individuals with isomyopia and emmetropia. Second, we wanted to examine whether differences in ocular characteristics between eyes in anisomyopia can directly predict the degree of binocular balance and its magnitude of improvement following optical correction.

## Materials and methods

### Participants

A total of 42 subjects participated in this study, including 15 anisomyopes (mean age ± SD: 24 ± 0.9 years), 15 isomyopes (23.7 ± 1.3 years) and 12 emmetropes (22.4 ± 3.2 years). All participants enrolled in this study had best corrected visual acuity of 0.00 log minimum angle of resolution logMAR or better with low astigmatism (<1.50D). All subjects had normal stereo acuity of 40–60 arcsec according to Yan’s Randot test (Birch et al., 2008; Cao et al., 2022). Fusion capability was evaluated using the worth 4-dot test at two viewing distances (33 cm and 6 m) to ensure intact fusion (both seeing 4 dots) (Bak et al., 2017). The clinical details of subjects are provided in Table 1. Refractive errors were obtained by subjective refraction without cycloplegia due to the age of subjects (young adults) (Tian et al., 2011). And all anisomyopic participants had a history of anisomyopia clinical diagnosis before our recruitment. Anisomyopia was defined as an interocular difference in myopic spherical equivalent refractive errors (SER = sphere + cylinder/2) of 1.5 D or more (Wang S. et al., 2020; Wang X. et al., 2020), isomyopia as less than 1.00 D difference of SER between the two eyes (Tian et al., 2011). Emmetropia was defined as +0.5D ≥ SER > −0.5D and had normal visual acuity (≤0.00 logMAR) (Ojaimi et al., 2005; Wang S. et al., 2020; Wang X. et al., 2020). Subjects were excluded if they had a history of eye diseases such as strabismus or amblyopia, a history of eye surgery, or high myopia (≥ 6.00D). Mile’s test was used to establish the dominant eye for psychophysical testing (Miles, 1930). Participants were asked to form a peephole using their both hands at arms’ length and view a visual target through the hole with both eyes open. Then they were asked to alternatively close each eye. When the dominant eye closed, the target disappeared or moved more. Visual acuity of each eye was tested using a Tumbling E acuity chart. All subjects were naïve to the purpose of the experiment and provided written informed consent before their enrollment. This study was approved by the Ethics Committee from Wenzhou Medical University (approval number: 2022-62-K-127-01) and followed the tenets of the Declaration of Helsinki.

**Table 1:** Clinical characteristics of the subjects.

Clinical characteristics (Mean ± SD)	Anisomyopia (*n* = 15)	Isomyopia (*n* = 15)	Emmetropia (*n* = 12)
Age (yrs)	24.00 ± 0.93	23.67 ± 1.35	22.42 ± 3.18
Visual acuity without correction (logMAR)			
Dominant eye	0.46 ± 0.36	0.52 ± 0.41	−0.08 ± 0.09
Non-dominant eye	0.56 ± 0.29	0.61 ± 0.39	−0.11 ± 0.08
Interocular visual acuity difference	0.54 ± 0.18	0.14 ± 0.20	0.03 ± 0.04
Stereoacuity (arcsecs)	49.33 ± 10.33	45.33 ± 9.15	43.33 ± 7.78
Spherical equivalent refractive errors (SER, diopter)			
Dominant eye	−2.12 ± 1.52	−2.54 ± 1.94	−0.08 ± 0.19
Non-dominant eye	−2.53 ± 1.07	−2.53 ± 1.81	0.20 ± 0.26
Interocular SER difference	1.91 ± 1.09	0.36 ± 0.27	0.34 ± 0.33
Axial length (mm)			
Dominant eye	23.77 ± 0.91	24.84 ± 1.12	23.94 ± 0.65
Non-dominant eye	23.91 ± 0.79	24.80 ± 1.06	23.82 ± 0.67
Interocular axial length difference	0.81 ± 0.27	0.16 ± 0.13	0.16 ± 0.14

For most values, their means and standard deviations are shown. Eye dominance was established using Mile’s test.

### Apparatus

On a MacBook Pro (13-in., 2017; Apple, Inc., Cupertino, CA, United States), we conducted the experiment using MATLAB R2016b (v9.1.0 MathWorks, Inc., Natick, MA, United States) with Psychtoolbox extension 3.0.14 (Brainard, 1997; Pelli, 1997). We dichoptically presented stimuli via gamma-corrected head-mounted goggles (GOOVIS, AMOLED display, NED Optics, Shenzhen, China) with a resolution of 1920 × 1,080 pixels, 41.6 pixels per screen degree, a refresh rate of 60 Hz and a maximal luminance of 150 cd/m^2^. In addition, the axial length of both eyes (the distance spanning from the front to its end of the eyeball) in all participants was measured using a non-invasive biometer (Lenstar LS 900, HAAG-STREIT AG, Switzerland) twice, and the average was used for subsequent data analysis.

### Stimuli and design

In this study, a binocular orientation combination task (Wang et al., 2019) was used to quantitatively measure binocular balance across a range of spatial frequency. Two sinusoidal gratings (size = 4.2° × 4.2°) covered by masks (size = 2.8° × 2.8°) were dichoptically shown by a head-mounted goggle. For instance, one grating with a positive orientation relative to the horizontal axis was shown to the right eye, and that with a negative orientation was shown to the left eye. The orientation differences between the two gratings were different for each of the four spatial frequencies (0.5, 1, 2 and 4 c/d); the orientations for both eyes were ± 4°, ±3.5°, ±3°, ±2.5° for 0.5, 1, 2, and 4 c/d. Data obtained at 8 c/d during the pilot experiment were unreliable and noisy because the psychometric function’s shape was not robust; so, we decided test at spatial frequency from 0.5 to 4 c/d. At higher spatial frequencies, the orientation difference was reduced to prevent mixed perception of the two gratings because the orientation tuning becomes narrower as a function of spatial frequency (Vidyasagar and Sigüenza, 1985). This is because the size of the gratings was fixed across high spatial frequencies, thereby introducing more cycles. In other words, the orientation difference at each spatial frequency was selected so that the likelihood of inducing mixed perception (superimposed or piecemeal) would be minimized. The grating’s size was fixed across spatial frequencies because we had realized that subjects could not perform the task reliably if we had kept the number of cycles constant rather than the size. For most subjects, seven contrast ratios of the two gratings were applied in each test block with base contrast at 0.28: 1/9, 1/3, 1/√3, 1, √3/1, 3/1, 9/1. However, for some subjects, the ratios and the base contrast had to be personalized because some failed to obtain a proper psychometric function using the standard values. There were 20 repetitions for each ratio and configuration; in one configuration, the dominant eye’s stimulus would have a positive orientation, whereas in the second configuration, it would have a negative orientation relative to the horizontal axis. Therefore, there were 140 trials total per test block. The contrast of gratings shown to the DE was base contrast×√ (contrast ratio), whereas the contrast presented to NDE was base contrast/√ (contrast ratio). Prior to each test block, the subjects confirmed that they were able to see the grating in each eye at all contrast ratios, experimental conditions (with and without optical correction) and spatial frequencies, confirming that the contrasts of the gratings were detectable at all times. The order of experimental conditions (spatial frequency and optical correction) was randomized across test blocks, and the order of contrast ratios and configurations was randomized during each test block.

### Experimental procedure

**Figure 1:**
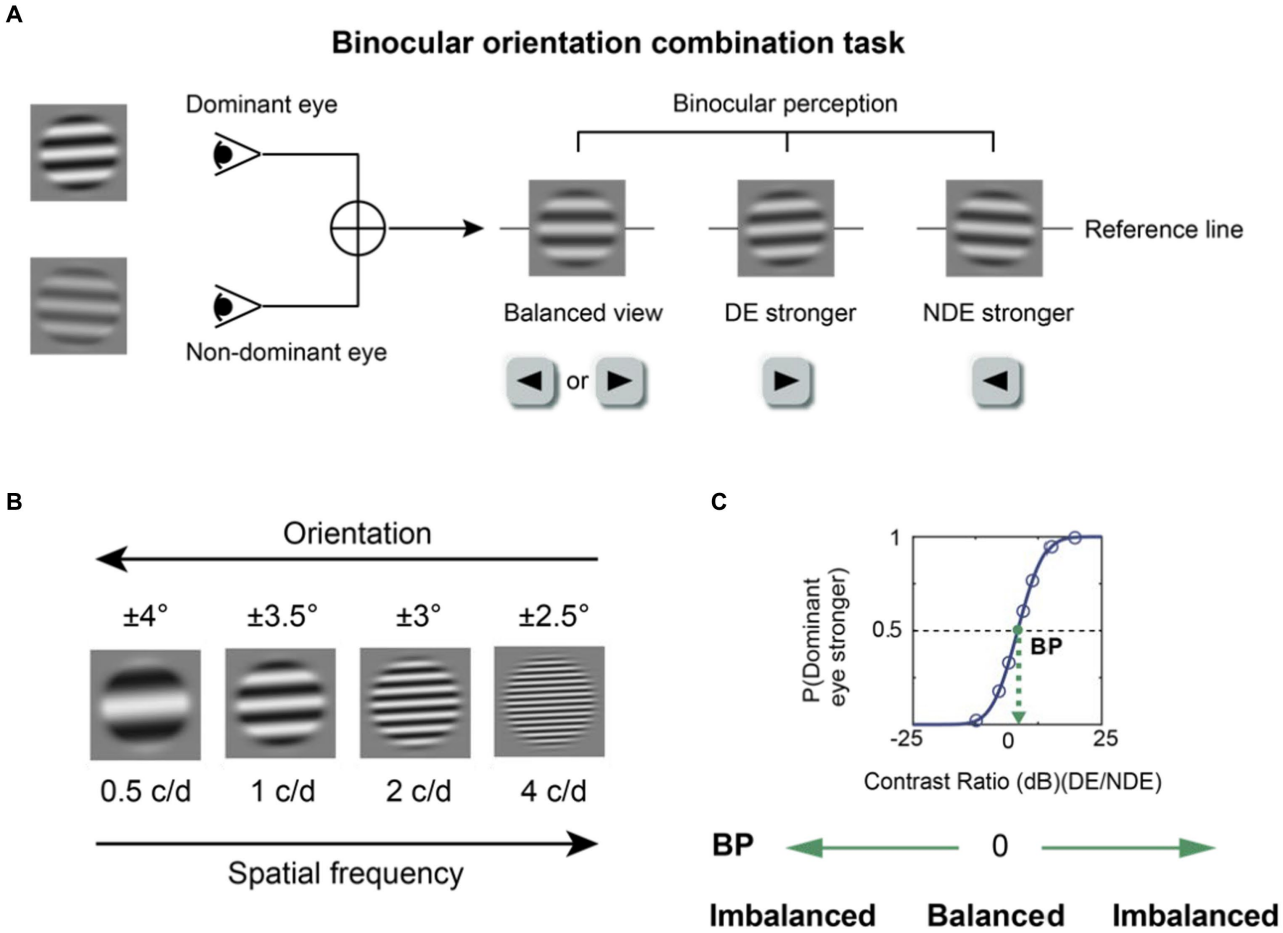
The binocular orientation combination task. **(A)** The visual stimuli. Two sinusoidal gratings with different contrasts and opposite orientations were dichoptically presented. The binocularly perceived grating would have an orientation of 0^o^ when there was a perfect binocular balance. The fused grating would be biased toward the orientation of the grating presented to one eye if that eye was more perceptually dominant. Participants were instructed to press the left or right key according to the orientation they perceived. DE, dominant eye; NDE, non-dominant eye. **(B)** Four spatial frequencies (0.5, 1, 2, 4 c/d) were tested. At a higher spatial frequency, a lower orientation difference was set between the two eyes to prevent mixed perception of the two gratings. **(C)** An illustration of the psychometric function. A cumulative logistic distribution function was used to fit the points into a psychometric function and estimate the balance point (shown by the green dot), which is the contrast ratio where two eyes contribute equally to binocular vision. The y-axis denotes the probability of the dominant eye being more perceptually prevalent.

We used a binocular orientation combination task (Wang et al., 2019) to quantitatively measure binocular balance (Figure 1A). The gratings were presented using a head-mounted goggle to ensure that the subjects could see the grating in each eye at all contrast levels. Every test block included two parts. Initially, the subjects were asked to complete a calibration trial in which they aligned the coordinates of a dichoptic cross to ensure perfect fusion. After the calibration, the subjects were asked to proceed by pressing the space bar on the keyboard so that the test phase could begin, where the two sinusoidal gratings appeared to the eyes dichoptically. Subjects were asked to report the binocularly perceived orientation of the fused gratings with the keyboard by either pressing the left or right key after the stimulus presentation. They were asked to guess the orientation when the fused grating had an orientation of 0^o^. The gratings were shown for a brief duration of 0.75 s to prevent the onset of rivalry from one percept to another during the stimulus presentation. Otherwise, if the stimulus duration was indefinitely long, the dominant percept could switch over time due to rivalry. The contrast level of the grating was increased from zero to its peak contrast level (set by the contrast ratio) for the first 0.25 s, then it remained at its peak for the next 0.25 s, then it decreased to 0 during the next 0.25 s based on a half-period of a sinewave function. A pixelated binary noise frame was presented around the stimuli throughout the task to facilitate fusion. After the subject responded, the next trial of the test phase would follow. Subjects spent about 3 min to complete a test block.

Emmetropic observers performed the visual task without optical correction because they had minimal refractive error. However, those with anisomyopia and isomyopia performed the visual task with and without optical correction using contact lenses at each spatial frequency. Each eye was fitted with a spherical soft contact lens (Bausch + Lomb®) based on the spherical equivalent refraction after vertexing to the corneal plane for participants with low astigmatism (<1.50D) (Moore et al., 2017). After optical correction, they had a best-corrected visual acuity of 0.00 logMAR or better. After they wore the contact lenses that produced the best optically corrected visual acuity, they were asked to adapt for 20 min, and then begin the psychophysical task. Subjects were tested once at each spatial frequency and in each optical state. It took about 1 h for each subject to complete the whole experiment.

### Data analysis

The balance point was measured from each test block using the orientation combination test. To do so, we had to convert the responses from the subjects who reported the binocularly perceived direction of the fused grating into a set of probability values where the dominant eye prevailed. To begin with, the contrast ratio was converted into log scales so that symmetry could be achieved:


$$\alpha_{dB}=20\times \log_{10}\left(\alpha_{ratio}\right)$$

where the contrast ratio can be expressed as:


$$\alpha_{ratio}=\frac{\alpha_{DE}}{\alpha_{NDE}}.$$

With $\alpha_{dB}$, the seven contrast ratios were equally spaced apart along the x-axis of the psychometric function, whose y-axis was the probability of the observer seeing the direction of the fused grating as that of the dominant eye. After we translated the responses into a set of probability values at the seven contrast ratios, we fitted the data using the Palamedes Toolbox (Prins and Kingdom, 2018). Specifically, the data were fitted with a cumulative logistic distribution function to estimate the contrast ratio where the two eyes would contribute equally to binocular vision. This contrast ratio is also referred to as the balance point (BP). As in previous studies (Mao et al., 2020; Chen et al., 2021), we converted the BPs into their absolute values (|BP|) to capture the absolute degree of binocular imbalance. The higher the|BP|, the larger the binocular imbalance. Furthermore, we converted the raw data BP into rBP (i.e., rectified BP) which is an index that is related to the refractive error of each eye. rBP equals BP if the dominant eye is less myopic or for emmetropia without no refractive errors, and (−1) × BP if the opposite is true. Thus, a positive rBP indicates that the eye with more myopic is more perceptually dominant and a negative rBP denotes that the eye with less myopic is more perceptually dominant.

Data were analyzed and visualized using R software (Min and Zhou, 2021). To better demonstrate the effect of spatial frequency on binocular balance, we plotted the slopes from the linear regression analysis between individuals’ |BP| or rBP and log_2_SF; this is also referred to as the dependence of binocular balance on spatial frequency throughout the paper. We also plotted the AUCs computed from individuals’|BP|or rBP as a function of log_2_SF to represent integrated binocular imbalance. The normality of data was checked using the Shapiro–Wilk test. When the data were not normally distributed, we performed square root transformation of the data to achieve a normal distribution. We used an analysis of variance (ANOVA) and then performed a pairwise post-hoc comparison test if necessary. An adjusted value of p of 0.05 was set as the criterion for statistical significance.

## Results

### Binocular balance in different refractive states

**Figure 2:**
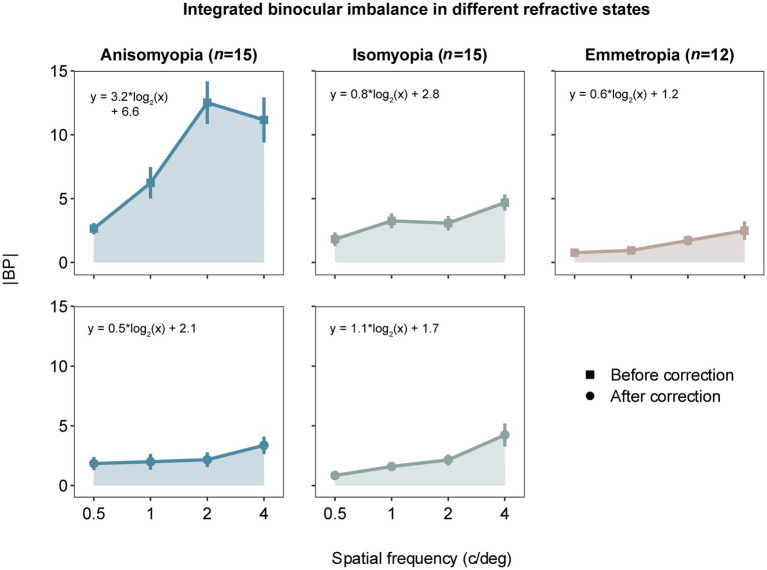
Absolute balance points (|BP|) as a function of spatial frequency in observers with anisomyopia (*n* = 15), isomyopia (*n* = 15) and emmetropia (*n* = 12). Blue points represent anisomyopes, green points isomyopes, peach points emmetropes. The square points show the corrected state, the rounded points the uncorrected state. Error bars represent standard errors. The larger the |BP|, the larger the binocular imbalance.

To begin with, we computed |BP| (i.e., magnitude of binocular imbalance) at each spatial frequency from all subjects and plot AUC as an index of integrated binocular imbalance. The larger the |BP|, the larger the binocular imbalance. According to Figure 2, we can observe that imbalance’s magnitude increases as a function of spatial frequency, especially in optically uncorrected individuals with anisomyopia. A three-way ANOVA (within-subject factors: spatial frequency and optical state; between-subject factor: subject group) revealed that there was a significant main effect of spatial frequency on binocular balance for isomyopia and emmetropia groups and uncorrected anisomyopia (*p’s* ≤ 0.03), verifying our initial observation. Moreover, there was a significant main effect of subject group when there was no optical correction (*F* (2,39) = 26.5, *p* < 0.0001, η^2^ = 0.406). However, after optical correction, no significant difference in |BP|was found among the three groups (*F* (2,39) = 0.7, *p* = 0.503, η^2^ = 0.021). We found that optical correction significantly lowered |BP|in anisomyopia at 1, 2, and 4 c/d, as well as in isomyopia at 0.5 and 1 c/d (*p’s* < 0.05). The analysis also revealed that there was a significant two-way interaction between spatial frequency and the optical state in anisomyopia (*F* (3,42) = 8.48, *p* = 0.00016, η^2^ = 0.112), as well as a significant three-way interaction among spatial frequency, optical state and subject groups (*F* (6,117) = 4.975, *p* < 0.0002, η^2^ = 0.048). The two-way interaction shows that the dependence of |BP|on spatial frequency is different between before and after optical correction in anisomyopia. The three-way interaction signals that there is a differential effect of spatial frequency on binocular balance across different optical states and subject groups.

In addition, we performed post-hoc pairwise comparisons to gain detailed insights about the data. First, |BP| was significantly different between each pair of the groups when there was no optical correction. Specifically, |BP| was found to be significantly different between anisomyopia and isomyopia at 1c/d, 2 c/d and 4 c/d (*p’s* ≤ 0.015). Moreover, |BP| was vastly different between anisomyopia and emmetropia at all spatial frequencies (*p’s* < 0.011). Furthermore, |BP| was notably different between isomyopia and emmetropia at 1 c/d (*p* = 0.0166). Also, in anisomyopia before optical correction, |BP| was significantly different between 0.5 c/d and the other three spatial frequencies, as well as between 1 c/d and 2 c/d, demonstrating that the imbalance significantly exacerbated as a function of spatial frequency. |BP| was significantly different between 0.5 c/d and 4 c/d in uncorrected isomyopia (*p* = 0.008). In isomyopia after optical correction, |BP| was found to be different between 4 c/d and the other three spatial frequencies, and between 0.5 c/d and 2 c/d (*P’s* ≤ 0.031). Surprisingly, no significant difference was found among three groups after optical correction. To summarize, |BP| was notably high in anisomyopia especially at 1c/d, 2 c/d and 4 c/d with a statistical significance, and |BP| was higher in isomyopia than in emmetropia at 1 c/d. |BP| increased significantly as a function of spatial frequency in almost all groups but not in corrected anisomyopia. This dependence on spatial frequency in anisomyopia was eliminated with optical correction using contact lenses.

**Figure 3:**
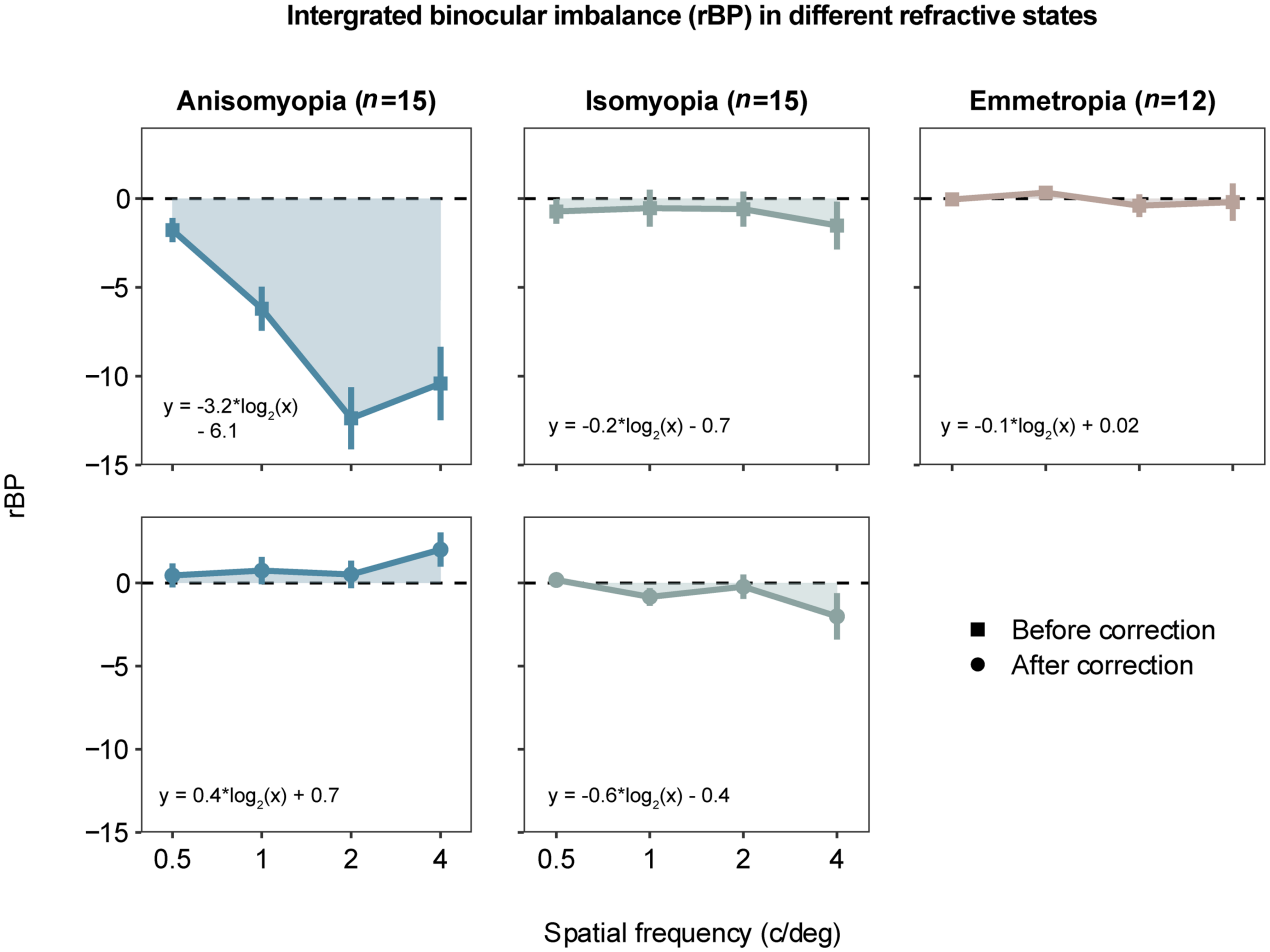
Area under a curve (AUC) computed by averaged rectified balance points (rBP) as a function of spatial frequency in different refractive states. The data are similarly presented as in Figure 2. A negative rBP indicates that the eye with less myopia is more perceptually dominant.

To investigate whether the dominant eye that drives the imbalance was more or less myopic, we also computed rBP by converting BP based on each eye’s spherical equivalent refraction. rBP as a function of spatial frequency is plotted for all three subject groups in Figure 3. After confirming that the data of rBP were normally distributed, we performed a three-way ANOVA, which showed a significant three-way interaction (*F* (3.97,77.47) = 9.549, *p* < 0.0001, η^2^ = 0.082). The analysis also revealed a two-way interaction between spatial frequency and the optical state in anisomyopia (*F* (2.01,28.1) =17.6, *p* < 0.0001, η^2^ = 0.182), as well as a significant main effect of spatial frequency in uncorrected anisomyopic observers (*F* (2.18,30.5) =17.4, *p* < 0.0001, η^2^ = 0.338). In addition, we observed a significant main effect of optical correction in anisomyopia as shown by the significant reduction of imbalance after optical correction at all spatial frequencies in Figure 3 (*p’s* ≤ 0.001). Moreover, post-hoc pairwise comparisons indicated that before optical correction rBP was significantly different between anisomyopia and isomyopia and between anisomyopia and emmetropia at 1 c/d, 2 c/d, and 4 c/d (*p*’s < 0.0008). rBP in uncorrected anisomyopia observers was quite different between 0.5 c/d and the other three spatial frequencies, and it was so between 1 c/d and 2 c/d (*P’s* ≤ 0.003). And rBP at 0.5 c/d was different from at 1 c/d in corrected isomyopia (*p* = 0.042). Together, these statistical results show that binocular balance of anisomyopia without optical correction is significantly in favor of the eye with a shorter axial length, especially from a spatial frequency of 1 c/d. However, the balance of isomyopia seems to be relatively intact before optical correction. Also, we found no significant difference in rBP among the three groups after optical correction, indicating that binocular imbalance in anisomyopia may be comparable to that in isomyopia and emmetropia.

**Figure 4:**
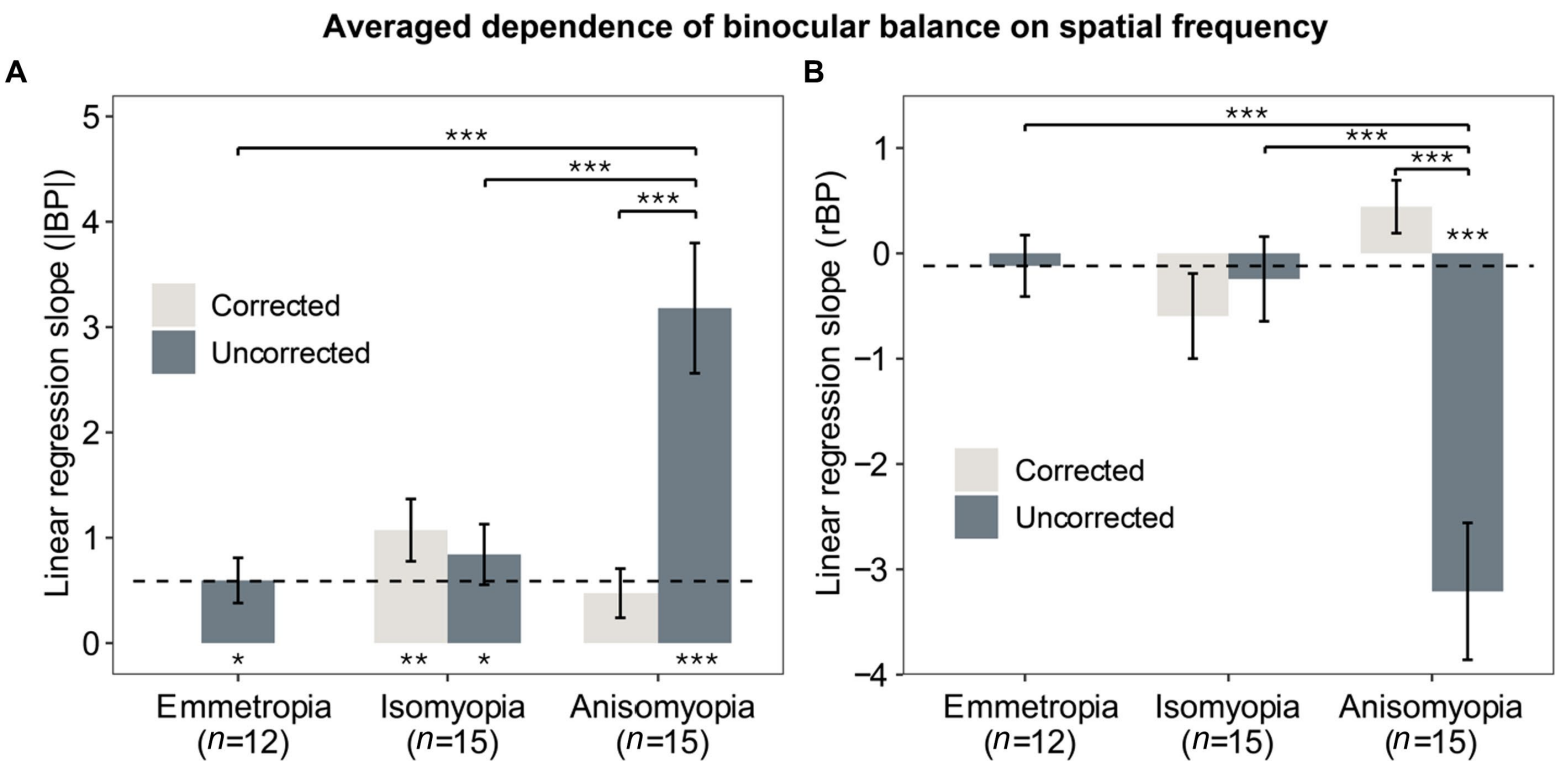
Averaged dependence of binocular imbalance on spatial frequency. **(A)** Slopes from linear regression function where the x-axis was the spatial frequency (log_2_SF) and y-axis was |BP|. **(B)** Slopes from linear regression function where the x-axis was the spatial frequency (log_2_SF) and y-axis was rBP. Light gray bars represent data after optical correction, dark blue bars data before optical correction. The dashed line denotes the averaged results of emmetropes. The * below or above the bars indicates statistical significance from one-sample *t*-test (in relation to 0): *p < 0.05, **p < 0.01, ***p < 0.001. Error bars show standard errors.

Previously, we separately analyzed binocular balance at each spatial frequency. Alternatively, binocular balance (y-axis) across a range of spatial frequency (x-axis) can be summarized as slopes from the best-fit linear regression, which captures the dependence of binocular balance on spatial frequency; the more the slope deviates from 0, the more severe the imbalance across spatial frequency. To compute the slope, we converted the spatial frequency into log2 units so that each level of spatial frequency was equally spaced along the x-axis. First, slopes based on |BP| were analyzed with a two-way mixed ANOVA (within-subject factor: optical state; between-subject factor: subject group), which showed that there was a significant interaction (*F* (2,39) = 12.133, *p* < 0.0001, η^2^ = 0.218) and a notable main effect of optical correction in anisomyopia (*F* (1,14) = 18.7, *p* = 0.0014, η^2^ = 0.375) and a significant main effect of subject group before optical correction (*F* (2,39) = 10.9, *p* = 0.0003, η^2^ = 0.36). However, no significant differences were found among the three groups. A post-hoc pairwise comparison reported that there was a significant difference between uncorrected anisomyopia and the other two groups (optically uncorrected; see Figure 4A) (*p’s* < 0.0011). Our result indicates that optical correction alleviates the dependence of binocular balance on spatial frequency more so in anisomyopia than in isomyopia (*F* (1,14) = 0.262, *p* = 1, η^2^ = 0.011).

Next, we also performed two-way mixed ANOVA for slopes based on rBP and found a significant interaction (*F* (2,39) = 13.687, *p* < 0.0001, η^2^ = 0.272). There were also significant effects of group and optical correction (*p’s* < 0.0003). Post-hoc pairwise comparisons with Bonferroni correction showed that there were significant differences on slopes based on rBP between anisomyopia and the other two groups (*p’s* < 0.0004). Finally, we compared slopes with 0 using a one-sample t-test to assess whether the dependence was significantly different from 0. These results are shown in Figures 4A,B in the form of asterisks below the bars. It shows that the slope based on |BP| was nearly flat for corrected anisomyopes; there was no significant difference between their slopes and 0 (*p* = 0.062), whereas all other groups had slopes (|BP|) that were significantly different than 0 (*p’s* ≤ 0.011). However, slopes based on rBP were significant different than 0 only in uncorrected anisomyopia. Together, the findings show that the dependence of binocular imbalance on spatial frequency is significantly worse in uncorrected anisomyopia than the other two groups. Also, there was no significant difference in the slopes among the three groups after optical correction, showing that the dependence on spatial frequency was similar in different refractive groups.

**Figure 5:**
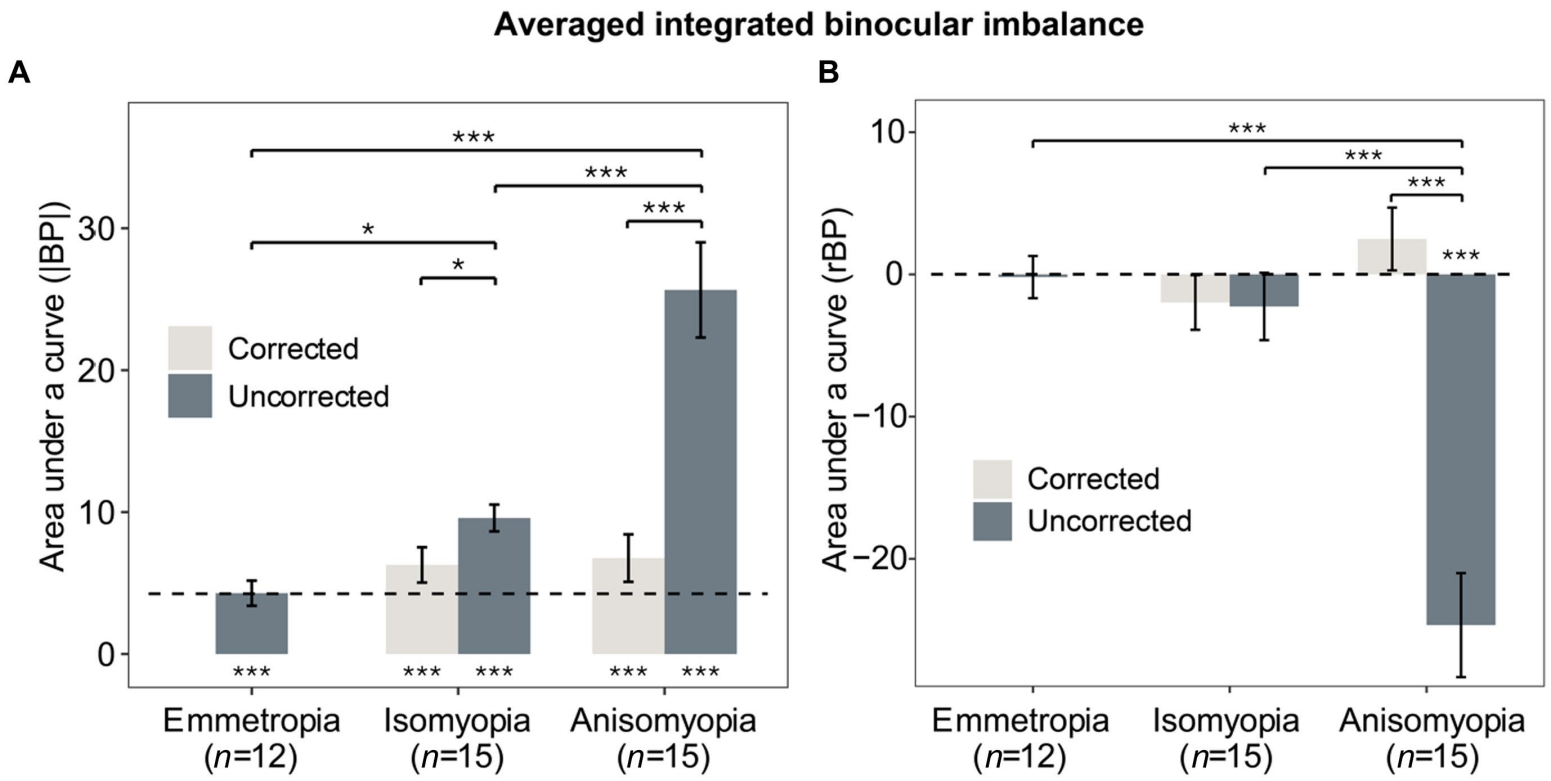
Averaged integrated binocular imbalance. **(A)** Mean AUCs of individuals’ |BP| as a function of log_2_SF for the three groups with and without correction. **(B)** Mean AUCs of individuals’ rBP as a function of log_2_SF for the three groups with and without correction. Gray bars represent corrected states, blue bars represent uncorrected states. The dashed line denotes the averaged results of emmetropes. The * below or above the bars indicates the significance between slope or AUC and 0. *p < 0.05, **p < 0.01, ***p < 0.001. Error bars show standard errors.

To further examine binocular imbalance, we computed area under a curve (AUC) of individuals’ |BP| and rBP as a function of log_2_ (SF). The greater the AUC based on |BP|, the larger the integrated binocular imbalance. As for the AUC based on rBP, it would be positive if the more myopic eye was dominant, but it would be more negative if the less myopic eye was more dominant. First, AUC based on |BP| was analyzed using a two-way mixed ANOVA (within-subject factor: state; between-subject factor: group), which showed a significant interaction (*F* (2,39) = 12.158, *p* < 0.0001, η^2^ = 0.241). AUCs (|BP|) in anisomyopia were quite different before and after optical correction (*p* < 0.0001). Results from post-hoc pairwise comparisons are shown in the form of asterisks in Figure 5A. Notably, both isomyopes and anisomyopes showed significant binocular improvement after optical correction because the AUCs decreased. However, there was no difference among the three groups after optical correction.

Next, AUC based on rBP was analyzed in a similar fashion using a two-way mixed ANOVA, which revealed a significant interaction (*F* (2,39) = 25.996, *p* < 0.0001, η^2^ = 0.356). Only anisomyopic observers showed binocular improvement after optical correction (see Figure 5B) according to a post-hoc pairwise comparison. Interestingly, in anisomyopes, AUC was less than 0 before optical correction but it was more than 0 after optical correction. Indeed, 1 anisomyope’s more myopic eye was more dominant before optical correction because the AUC was larger than 0 (not shown). The remaining 14 anisomyopes’ more myopic eye was less dominant. However, after optical correction, 7 anisomyopes’ more myopic eye (exactly half of our sample size) was more perceptually dominant after optical correction. Also, t-test showed no significant difference in SER between the dominant eye and the non-dominant eye (*p’s* > 0.19). This finding does not show that a certain eye is necessarily more dominant when both eyes have been corrected in anisomyopes. Moreover, a one-sample *t*-test showed that AUC based on rBP in corrected anisomyopes was not significantly different 0, showing that their binocular balance was very close to being normal.

### Relationship between perceptual balance and clinical characteristics

**Figure 6:**
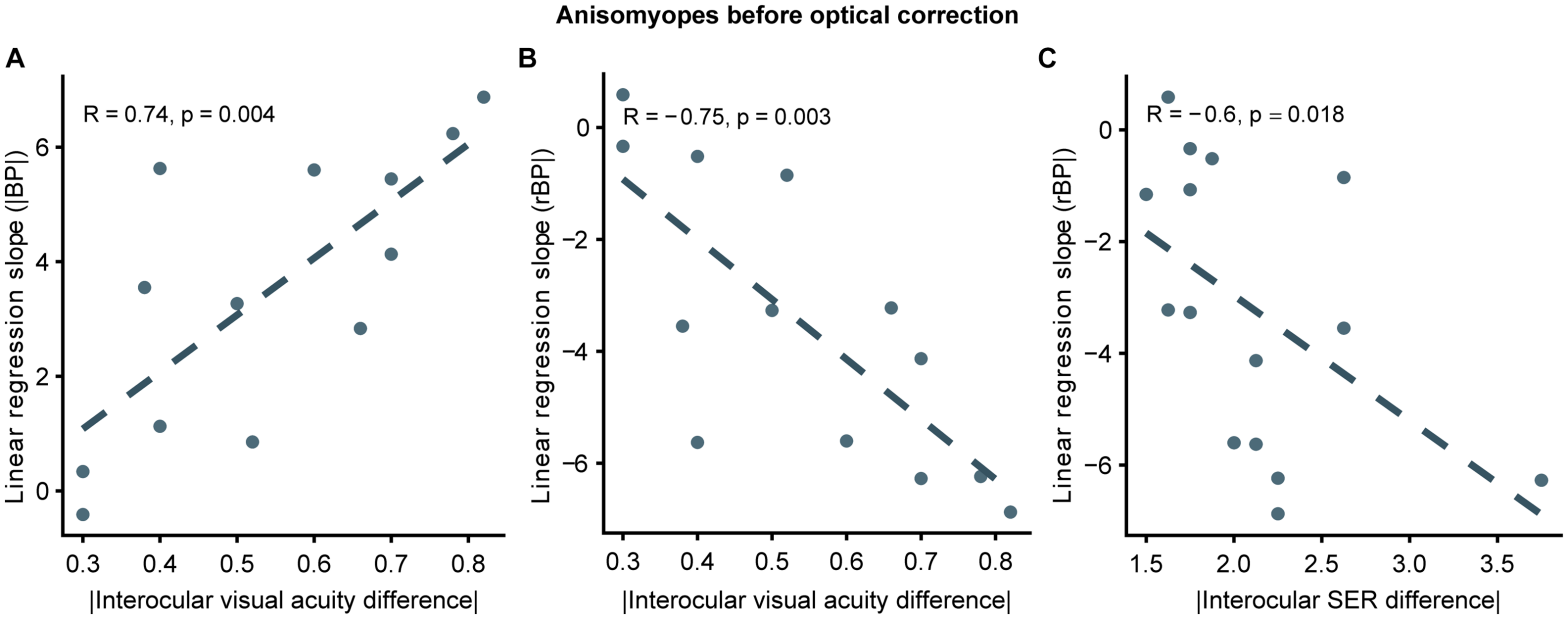
Correlation between slopes and interocular differences in ocular features in uncorrected anisomyopes. **(A)** Correlation between slopes computed from |BP| and absolute values of interocular visual acuity difference. **(B)** Correlation between slopes computed from rBP and absolute values of interocular visual acuity difference. **(C)** Correlation between slopes computed from rBP and absolute values of interocular SER difference. SER, spherical equivalent refraction. Dark blue points represent anisomyopic individuals [*n* = 13 in panels **(A, B)**; two subjects did not come for a follow-up session for visual acuity measurement]. The dotted lines are best-fit linear regressions from each scatter plot based on the correlation coecient.

As illustrated previously, we observed that binocular balance in anisomyopes was nearly normal after optical correction, demonstrating that ocular characteristics could determine the state of binocular imbalance. Therefore, we examined whether binocular imbalance in anisomyopic observers with any clinical characteristics. Pearson correlation analyzes revealed a significant association between the absolute interocular difference in the uncorrected visual acuity and the slopes (based on rBPs and |BP|) (*p’s* ≤ 0.004, |R|’s > 0.7; see Figures 6A,B). Also, we found that slopes based on rBP had a significant relationship with the absolute interocular SER difference according to a Spearman correlation analysis (*p* = 0.018, R = −0.6; see Figure 6C) However, interocular visual acuity difference had no significant association with binocular balance (slopes or AUCs) in anisomyopia after optical correction. Also, no significance was found between binocular balance and interocular axial length difference, the presence of long-term optical correction, subject age, or magnitude of anisomyopia both before and after optical correction. These findings indicate that interocular difference in visual acuity and SER could predict binocular imbalance before optical correction in anisomyopia, but there is no relationship between these clinical characteristics and binocular balance after correction.

**Figure 7:**
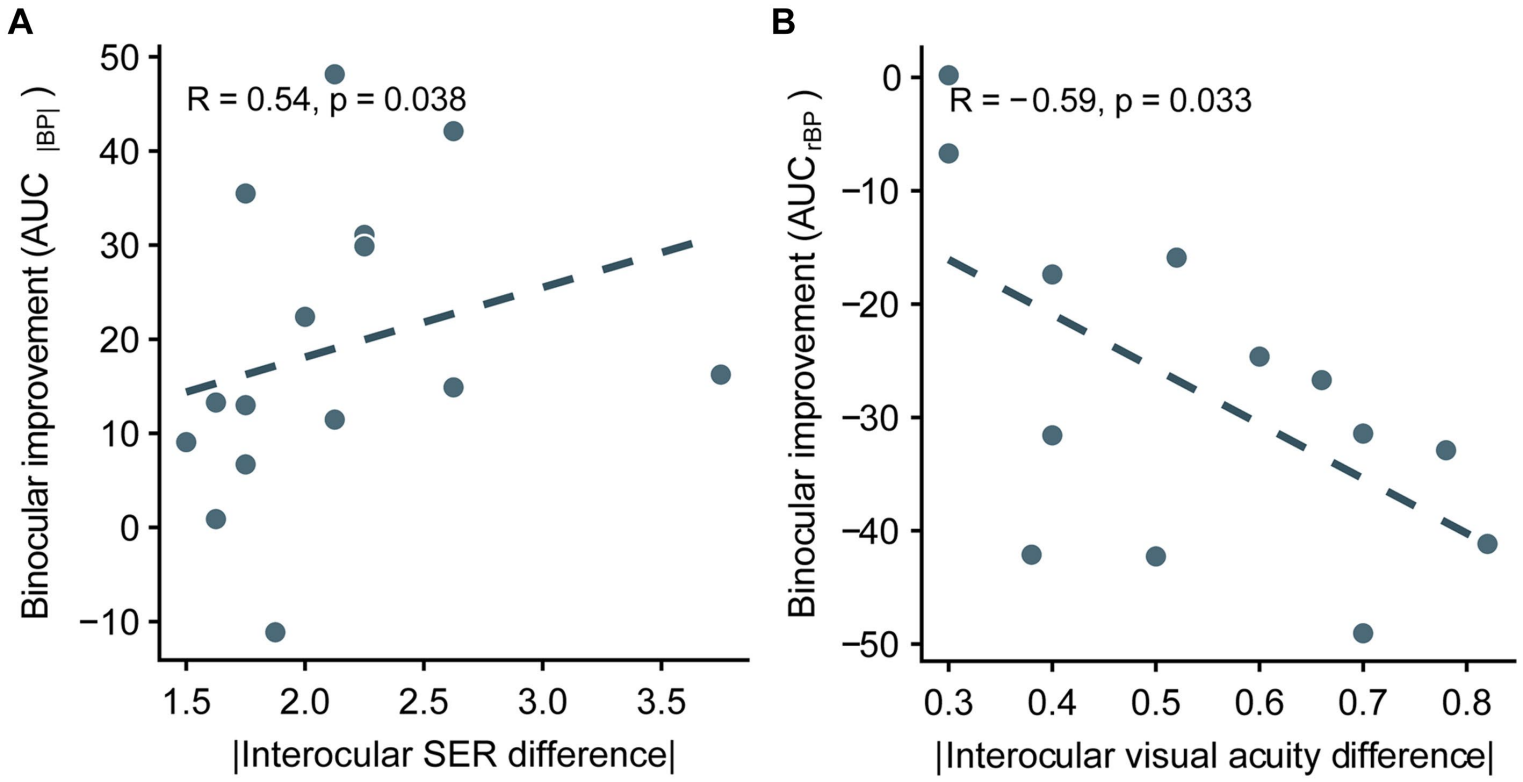
Correlation between the improvement of integrated binocular imbalance (the difference between AUC before optical correction and AUC after correction) from optical correction and the difference in ocular characteristics between two eyes in anisomyopia. **(A)** Correlation between the difference in AUCs computed from |BP| and absolute interocular SER difference. **(B)** Correlation between the difference in AUCs computed from rBP and absolute interocular visual acuity difference. SER, spherical equivalent refraction. The dashed lines are best-fit linear regressions from each scatter plot based on the correlation coecient.

Thus far, we have demonstrated that optical correction entirely relieves binocular imbalance in anisomyopia, suggesting that there is no residual uncorrectable balance from neural source. For this reason, we wanted to examine whether there would be relationship between improvement in binocular balance (the difference between AUC before optical correction and AUC after correction) following optical correction and the interocular difference in ocular characteristics in anisomyopic observers. The larger the difference in AUC_|BP|_ as shown in Figure 7A, the larger the binocular improvement. Conversely, the smaller the difference in AUC_rBP_ (see Figure 7B), the larger the binocular improvement. We found that the difference in AUC of rBP was significantly correlated with absolute interocular visual acuity difference, and the difference in AUC of |BP| was significantly correlated with the magnitude of anisomyopia (i.e., absolute interocular SER difference) (*p’s* ≤ 0.038, |R|’s > 0.5; see Figures 7A,B). However, we did not find a significant relationship between interocular differences in axial lengths and the difference either in AUC_|BP|_ or in AUC_rBP_ (*p’s* > 0.14). In sum, our result shows that the improvement of binocular imbalance from optical correction was associated with certain ocular factors, such as absolute interocular visual acuity difference and the magnitude of anisomyopia.

## Discussion

This study examined binocular balance in individuals with isomyopia and anisomyopia before and after optical correction with contact lenses. First, we found no significant difference in binocular imbalance among corrected anisomyopes, corrected isomyopes and uncorrected emmetropes, showing that binocular imbalance before optical correction was primarily due to ocular factors because it was entirely alleviated with optical correction. Second, the reduction of the areal measure following optical correction (i.e., improvement of binocular balance) was associated with absolute interocular difference in visual acuity and SER but not in axial length. These findings collectively suggest that binocular improvement after optical correction can be predicted based on certain ocular characteristics of anisometropia and that optical correction adequately relieves imbalance in anisomyopic observers.

Our findings indicating that binocular balance across spatial frequency in anisomyopia was quite similar with that in isomyopia and emmetropia are surprising because they demonstrate that binocular deficit from anisometropia occurs mainly due to optical factors. For example, we found that interocular difference in SER in anisomyopia predicted the improvement of sensory eye dominance following optical correction. However, Zhou et al. (2016) suggested that imbalance in anisometropia could be neuronally driven because anisometropes who had been previously corrected for 16 weeks still showed binocular imbalance at 1 c/deg. There are some possible reasons for the difference between their results and our findings. The previous study included observers with hyperopic anisometropia, which could exacerbate binocular visual functions (Lee et al., 2013; Hu et al., 2016). Also, spectacles rather than contact lens were used for optical correction during psychophysical measurement, potentially inducing greater aniseikonia and demand for suppression (Winn et al., 1988; South et al., 2019). However, whether aniseikonia is the cause of binocular imbalance in anisomyopes still needs to be verified by quantitative measurement of aniseikonia before and after optical correction in further studies. In addition, different paradigms could induce different results. Zhou et al. used a binocular phase combination task at one spatial frequency (1 c/deg), while we used a binocular orientation task which was reported to be more precise to evaluate binocular balance especially at high spatial frequency (Wang et al., 2019). Finally, observers had a more severe anisometropia (mean interocular spherical refraction >3D) in the study of Zhou et al. (2016), whereas anisomyopes in our sample had average anisometropia of 1.91D, which could be categorized as mild anisometropia whose occurrence rate is high (Wang S. et al., 2020; Wang X. et al., 2020). Although we observed no residual imbalance after optical correction (i.e., neuronal imbalance) in anisomyopes, the possibility that there could be a greater binocular imbalance in individuals with a more severe anisomyopia even after optical correction cannot be excluded.

As previously mentioned, binocular balance was found to be close to normal in anisomyopes after optical correction because the source of imbalance in binocular combination was mostly optical not neuronal. This indicates that differences in ocular characteristics, such as asymmetrical axial lengths, between eyes that are frequently observed in anisometropia (especially anisomyopia) can induce binocular imbalance, which we were able to alleviate with optical correction (Huynh et al., 2006; Tian et al., 2011). However, whether these differences in ocular characteristics between eyes directly predict the magnitude of sensory eye balance is unknown because most studies have qualitatively determined ocular dominance (i.e., which eye is more dominant) but have not quantitatively measured its magnitude (Cheng et al., 2004; Linke et al., 2011; Vincent et al., 2011). Hence, in this study, we quantitatively measured binocular balance in optically corrected anisomyopia to evaluate whether ocular characteristics in each individual could directly predict binocular imbalance at various spatial frequencies and its improvement following correction in anisomyopic observers, most of whom had mild anisometropia. Although, we did not find a strong correlation between binocular imbalance and its improvement following optical correction and interocular difference in axial length, we observed that binocular imbalance before optical correction and its improvement were significantly correlated with interocular differences in visual acuity as well as SER. In particular, our results indicate that interocular differences in visual acuity and SER can predict binocular imbalance in anisomyopia without optical correction.

Our findings also dispel the view that a certain eye tends to be more perceptually dominant when both eyes have been corrected in mild anisometropia. For example, before optical correction, the integrated balance (AUC based on rBP) favored the less myopic eye in 14/15 anisomyopes. This is expected because the more myopic eye has lower perceived contrast than the other eye before optical correction, thereby having less perceptual weight in binocular combination (Fahle, 1982). However, after optical correction, 8 anisomyopes showed ocular dominance in favor of the less myopic eye after optical correction, whereas the remaining half of the anisomyopic sample (*n* = 7) showed dominance in favor of the more myopic eye. Also, no clear difference in SER or axial length (Supplementary Figure S1) was found between the dominant eye and the non-dominant eye. These findings do not support the idea that one eye should be more particularly dominant after both eyes have been corrected in anisomyopia. These results are different from those in previous studies. For example, some studies have shown that the more dominant eye had a significantly longer axial length and hence more myopic (Cheng et al., 2004; Vincent et al., 2011). Other studies, however, have reported the opposite relationship (Linke et al., 2011; Ito et al., 2013). Nevertheless, our results showing no relationship between various clinical characteristics and the extent of sensory ocular dominance after correction, agree with those from some other studies (Chia et al., 2007; Eser et al., 2008; Yang et al., 2008) that have reported no association between qualitatively determined ocular dominance and interocular differences in ocular characteristics such as SER and axial lengths. Also, a longitudinal 2-year study (Yang et al., 2008), which examines the development of myopia in children, reports that the dominant eye does not necessarily become more myopic in children during visual development, demonstrating no robust relationship between ocular dominance and the refractive status of both eyes. Ideally, future studies should use quantitative methods to further explore the possible relationship between ocular dominance and asymmetric ocular characteristics in anisometropia using a longitudinal experimental design.

Another visual condition that has significant binocular imbalance is amblyopia, which is a neurodevelopmental disorder from abnormal visual experience in early life (Kiorpes and McKee, 1999; Birch, 2013). Whether the degree of binocular imbalance that is observed due to optical factors in anisomyopia is comparable to that of neuronal imbalance of amblyopia remains unknown. If they are similar, the way how the refractive error attenuates the more myopic eye’s weight in binocular fusion in anisomyopia can be comparable to how the fellow eye suppresses the poor eye in amblyopia. For this reason, we have compared our data from anisomyopes, isomyopes and emmetropes against previously published data from individuals with ongoing and treated amblyopia (see the Supplementary Material for more details). According to our analysis, we observed that imbalance gets noticeably worse at higher spatial frequencies more in optically corrected amblyopia and treated amblyopia (Supplementary Figure S2) than in uncorrected anisomyopes, indicating that the abnormal interocular suppression in amblyopia drives more binocular imbalance than differential blur from anisomyopes. The fact that neuronal imbalance in amblyopia behaves differently than optical imbalance in anisomyopia could be due to the fact that there are multiple sources could decrease the weight of the poor eye in binocular vision, such as an increased suppression by the fellow eye (Zhou et al., 2018) and higher internal noise of the poor eye (Reynaud and Min, 2023). Together, these findings indicate that disrupted binocular balance from asymmetrical refractive status in anisomyopes is not analogous to optically uncorrectable imbalance in amblyopic visual system.

This study has some limitations. First, most anisomyopic subjects in our sample had mild interocular difference in SER, so our findings may not be generalizable to those with severe anisomyopia. In addition, we used data from subjective refraction without cycloplegia. Future studies should investigate binocular visual functions of those with a wider range of anisomyopia. Also, 13 of the 15 anisomyopes we recruited had undergone spectacles correction for more than 2 years and all started correction after 14 years old. Longitudinal experiments should be taken to clarify the effect of long-term optical correction on binocular balance in anisomyopia.

In conclusion, binocular imbalance in corrected anisomyopes was found to be similar to that of corrected isomyopes and uncorrected emmetropes, demonstrating that there is only optical source that disrupts balance before optical correction but no neuronal source that causes uncorrectable residual imbalance in anisomyopia. Binocular improvement following optical correction was correlated with absolute interocular difference in visual acuity and SER but not in axial length in anisomyopia. Also, interocular differences in visual acuity and SER were associated with binocular imbalance in uncorrected anisomyopia. However, our findings do not show a clear relationship between clinical characteristics and binocular balance in corrected anisomyopia. They also do not support the idea that a certain eye is more perceptually dominant when both eyes have been optically corrected. Future studies should explore whether there can be a stronger quantitative relationship between clinical characteristics and binocular imbalance in individuals with more severe anisomyopia.

## Data availability statement

The raw data supporting the conclusions of this article will be made available by the authors, without undue reservation.

## Ethics statement

The studies involving humans were approved by the Ethics Committee from Wenzhou Medical University (approval number: 2022-62-K-127-01). The studies were conducted in accordance with the local legislation and institutional requirements. The participants provided their written informed consent to participate in this study.

## Author contributions

NJ: Conceptualization, Data curation, Formal analysis, Investigation, Methodology, Writing – original draft, Writing – review & editing, Validation, Visualization. YZ: Data curation, Formal analysis, Methodology, Conceptualization, Investigation, Project administration, Writing – review & editing. MC: Conceptualization, Data curation, Methodology, Formal analysis, Investigation, Project administration, Writing – review & editing. JZ: Conceptualization, Funding acquisition, Investigation, Methodology, Project administration, Resources, Supervision, Validation, Writing – review & editing. SM: Conceptualization, Formal analysis, Investigation, Methodology, Project administration, Supervision, Validation, Writing – review & editing, Funding acquisition, Resources, Software, Visualization, Writing – original draft.
