# Corrigendum: Binocular balance across spatial frequency in anisomyopia

**DOI:** 10.3389/fnins.2024.1457590

**Published:** 2024-07-23

**Authors:** Nan Jiang, Yang Zheng, Mengting Chen, Jiawei Zhou, Seung Hyun Min

**Affiliations:** School of Ophthalmology and Optometry, Affiliated Eye Hospital, State Key Laboratory of Ophthalmology, Optometry and Vision Science, Wenzhou Medical University, Wenzhou, China

**Keywords:** anisomyopia, binocular vision, contact lenses, axial length, visual acuity

In the published article, there were errors in Figures 6C, 7A. Although the x-axis indicated the absolute value of interocular SER (spherical equivalent refraction) difference, some points were negative. The correct versions of Figures 6, 7 are shown below. Figure captions and the associated statistical results are the same as before.

**Figure 6:**
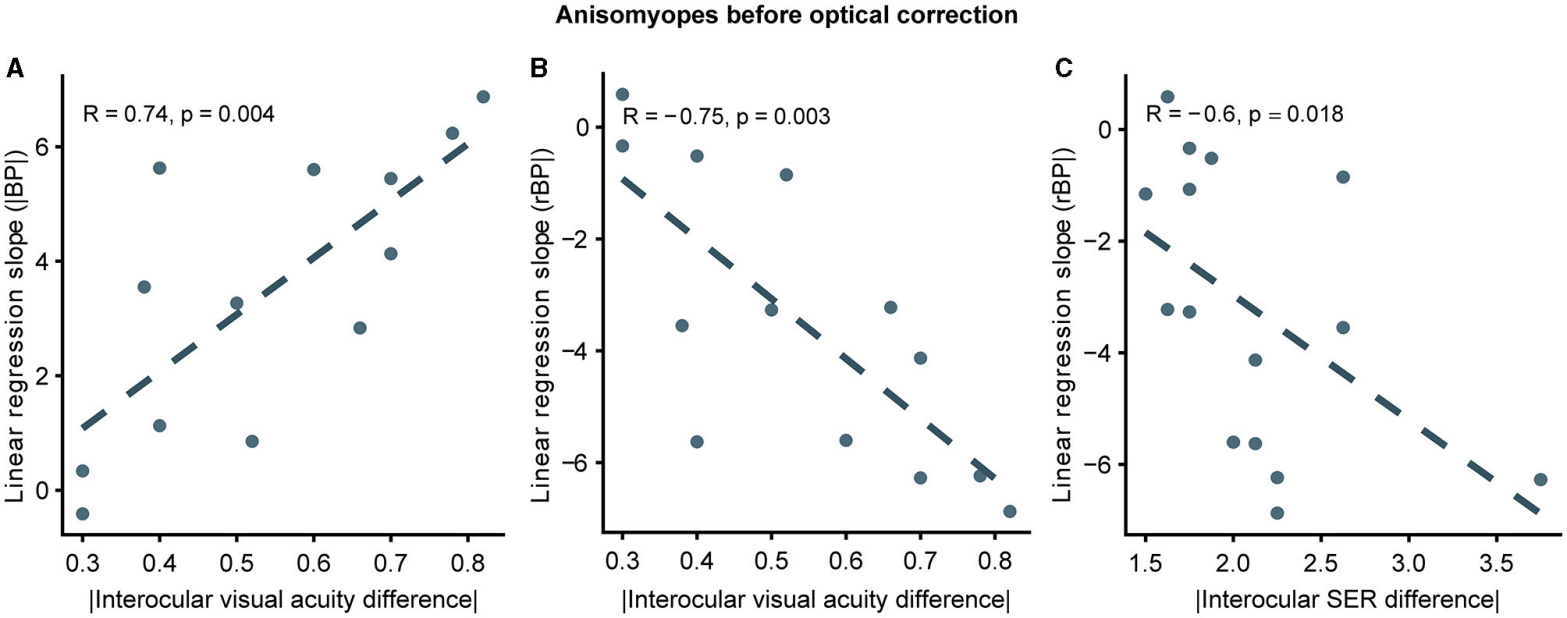
Correlation between slopes and interocular differences in ocular features in uncorrected anisomyopes. **(A)** Correlation between slopes computed from |BP| and absolute values of interocular visual acuity difference. **(B)** Correlation between slopes computed from rBP and absolute values of interocular visual acuity difference. **(C)** Correlation between slopes computed from rBP and absolute values of interocular SER difference. SER, spherical equivalent refraction. Dark blue points represent anisomyopic individuals [*n* = 13 in panels **(A, B)**; two subjects did not come for a follow-up session for visual acuity measurement]. The dotted lines are best-fit linear regressions from each scatter plot based on the correlation coefficient.

**Figure 7:**
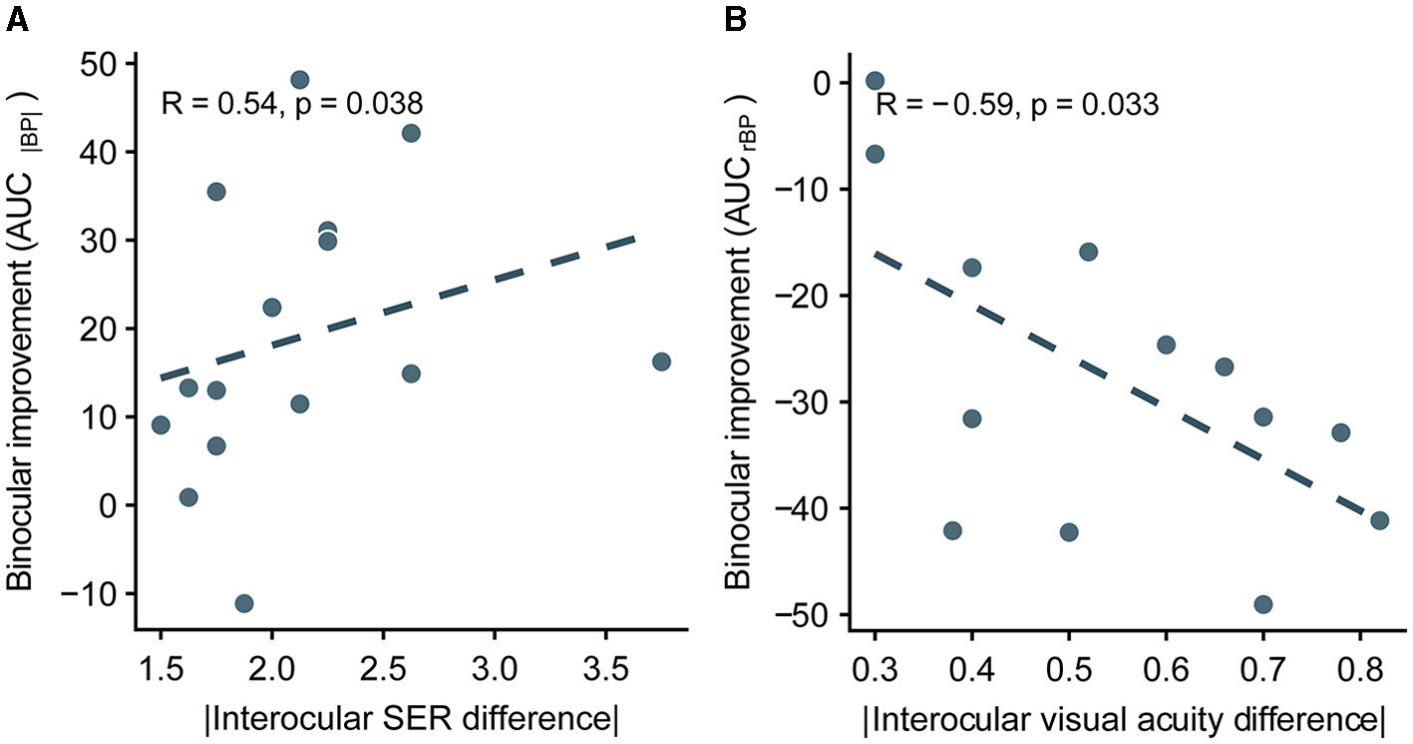
Correlation between the improvement of integrated binocular imbalance (the difference between AUC before optical correction and AUC after correction) from optical correction and the difference in ocular characteristics between two eyes in anisomyopia. **(A)** Correlation between the difference in AUCs computed from |BP| and absolute interocular SER difference. **(B)** Correlation between the difference in AUCs computed from rBP and absolute interocular visual acuity difference. SER, spherical equivalent refraction. The dashed lines are best-fit linear regressions from each scatter plot based on the correlation coefficient.

The authors apologize for this error and state that this does not change the scientific conclusions of the article in any way. The original article has been updated.

