# Supplementary material for: Binocular balance across spatial frequency in anisomyopia
Source: Front Neurosci. 2024 Jan 25;18:1349436. doi: 10.3389/fnins.2024.1349436 (PMC10850230; doi:10.3389/fnins.2024.1349436)
Supplement: Supplementary file 1 [file Data_Sheet_1.pdf]

Supplementary material

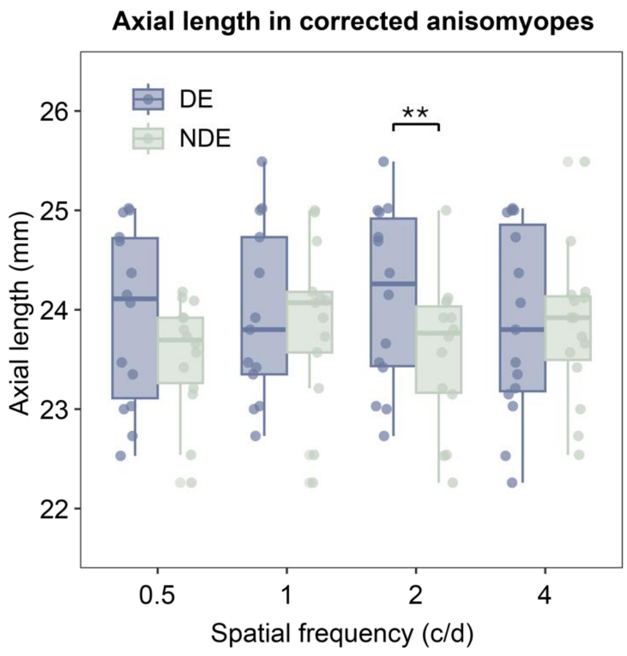

Figure S1. Boxplot of axial length between dominant and nondominant eyes in corrected anisomyopes at different spatial frequency. Purple represents the dominant eye, green represents the nondominant eye. Points are the axial length of individuals. The line in the middle of box means the median of the data. DE: dominant eye, NDE: nondominant eye. $^{**}P < 0.01$.

We found that the more myopic eye was more perceptually dominant in anisomyopes after optical correction. So, we examined whether there would be a difference in axial length between the dominant eye and non-dominant eye in corrected anisomyopes. Figure S1 shows that the axial length in the dominant eye is not significantly different from that in the non-dominant eye at most spatial frequencies (P's ≥ 0.119) except for 2 c/d ($P = 0.008$), revealing that axial length is not clearly correlated with eye dominance in anisomyopia.

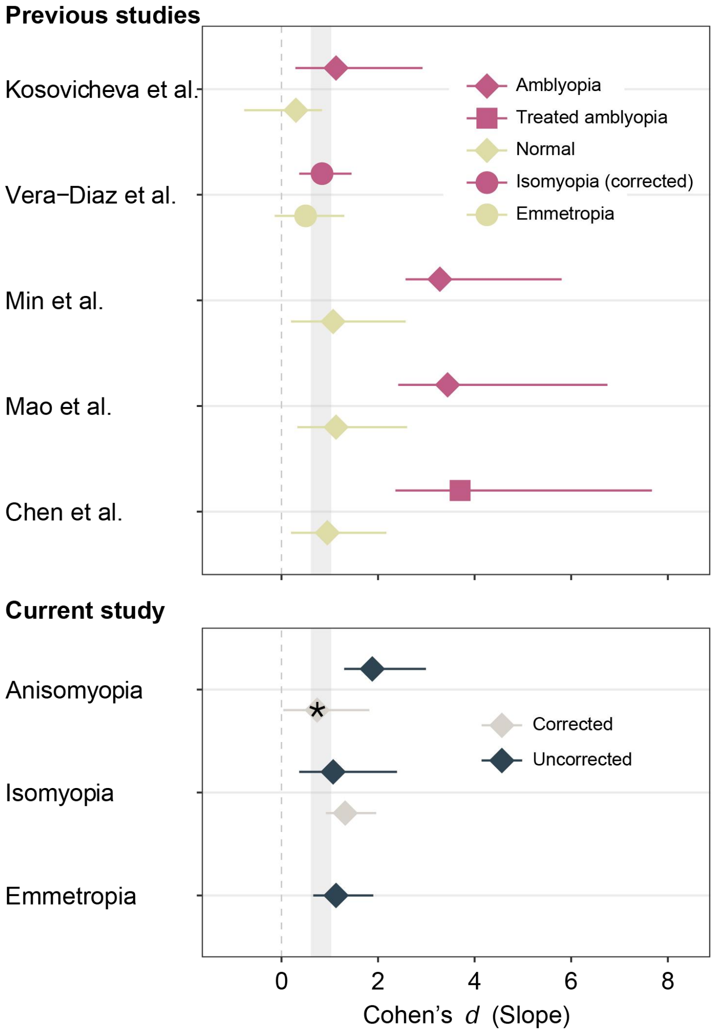

Figure S2. Meta-analysis is illustrated with a forest plot using data from current and previous studies(Kosovicheva et al., 2019; Vera-Diaz et al., 2018; Min et al., 2022; Mao et al., 2020; Chen et al., 2021). The effect size (Cohen's *d*) was computed from slopes that captured the dependence of absolute degree of binocular balance across spatial frequency. A positive effect size indicates that the balance gets worse at a higher spatial frequency. A negative effect size indicates that the balance gets worse at a lower spatial frequency. According to the previous studies, normal controls were those with normal or corrected-to-normal vision (≤ 0.0 logMAR); amblyopes were those with a difference in visual acuity of at least 0.20 logMAR; the treated amblyopes were those who had their previously amblyopic eye with 0.1 logMAR or better and an interocular difference in visual acuity less than 0.20 logMAR. In Vera-Diaz's study, emmetropia was defined as SE +0.75D~-0.25D, and isomyopia was defined as SE -0.75D~-8.00D with ≤1.00D anisometropia and ≤1.5D astigmatism; subjects with isomyopia were optically corrected with spectacles during the test. The error bars indicate 95% confidence intervals of the effect sizes. Dashed line is a reference for effect size at 0. The asterisk (*) denotes that the confidence interval does not overlap with 0 for a specific dataset that has a very close distance to 0. The grey shade indicates the 95% confidence interval of effect size of all normal (and emmetropic) observers pooled from the six studies (including the current one).

We collated datasets from various behavioral studies that have used similar psychophysical methods (Vera-Diaz et al., 2018; Kosovicheva et al., 2019; Mao et al., 2020; Chen et al., 2021; Min et al., 2022) with our empirical data to see if imbalances across different visual impairments were similar. The data from five previous studies (total subjects = 117) were extracted and compared against our data (42 subjects).

Kosovicheva et al. and Vera-Diaz et al. used a dichoptic letter chart to test binocular balance by eliciting binocular rivalry, whereas the others including the current study used binocular orientation task with some minor differences in the design (Mao et al., 2020; Chen et al., 2021; Min et al., 2022), such as the contrast ratios, the number of trials and the number of cycles in the stimuli. We only used data from experiments where subjects were asked to see a static (i.e., flicker rate = 0 Hz) dichoptically presented stimulus and report whether it resembled the stimulus shown to the dominant eye or that the other eye. We summarized the data of binocular balance at multiple spatial frequencies by computing the linear regression slope, which captures the dependence of binocular balance on spatial frequency (Kwon et al., 2015; Mao et al., 2020), and then converted the data into Cohen's *d* given that the units of the data from different studies were not identical. A larger effect size indicates a more significant difference between the slope (y-axis: absolute values of the balance point; x-axis: logarithmically spaced spatial frequency) and 0. The larger the effect size, the larger the dependence of binocular balance on spatial frequency relative to 0. The 95% confidence interval of the effect size was computed using a non-parametric bootstrapping procedure with 1000 iterations of resampling with replacement (Efron, 1988), and it capture the range of the effect size where the true slope from the population resides with a 95% probability.

From the forest plot, we can make several important observations. First, the effect sizes from amblyopes and treated amblyopes were much larger. The cause for the dependence of imbalance on spatial frequency is most likely neural since treated amblyopes have intact visual acuity (i.e., 0.1 logMAR or better). Therefore, it seems that the neural origin of binocular imbalance is quite potent and significant. In addition, according to our data, optically uncorrected anisomyopes showed a larger effect size than optical corrected ones but it still smaller than those from treated and untreated amblyopes, indicating that the ocular source of imbalance less potent than the neural imbalance. Imbalance from ocular, but not neural, source was reduced to near 0 through optical correction (see Figure S2; corrected anisomyopia vs. treated amblyopia). The effect sizes of normal controls, emmetropes, corrected and uncorrected isomyopes as well as corrected anisomyopes were quite similar. These results demonstrate that there are both ocular and neural causes for binocular imbalance, and that neural cause can induce a significantly larger and optically uncorrectable imbalance. Surprisingly, the confidence interval of all normal (optically corrected) and emmetropic subjects (n = 120) pooled from six studies does not overlap with 0, indicating that the fact that imbalance gets slightly worse at a higher spatial frequency is in fact common even in the visually intact population. Furthermore, we observed that the effects of optical correction using spectacles and contact lenses were comparable on binocular imbalance as shown by the similar effect sizes of optically corrected anisomyopia (our study) and isomyopia (Vera-Diaz et al. in Figure 7), respectively. This finding is surprising given the fact that spectacles, rather than contact lenses, have more chance to induce aniseikonia, which can disrupt interocular suppression (Winn et al., 1988; South et al., 2019). In sum, our analysis revealed that there are ocular and neural origins of perceptual imbalance in binocular vision, and that only the former is optically correctible.

Nevertheless, it is important to point out that there was a difference in effect sizes from between binocular rivalry and orientation tasks. For instance, the effect size of amblyopia in Kosovicheva et al. (binocular rivalry) was lower than those from Mao et al. and Min et al. (binocular orientation). However, we see that the confidence interval was much wider in amblyopia (Kosovicheva et al.) than that in myopia (Vera-Diaz et al.) even if these two were obtained from binocular rivalry tasks, indicating that the effect size of amblyopia

was still larger. Also, despite the similarity in the visual tasks, the effect size for anisomyopia from our data was markedly smaller than those from amblyopia in the data of Mao et al. and Min et al., suggesting that the neural imbalance in amblyopia was much worse than the ocular imbalance in anisomyopia.

References

Chen, S., Min, S. H., Cheng, Z., Xiong, Y., Yu, X., Wei, L., et al. (2021). Binocular visual deficits at mid to high spatial frequency in treated amblyopes. *iScience* 24, 102727. doi: 10.1016/j.isci.2021.102727.

Efron, B. (1988). Logistic Regression, Survival Analysis, and the Kaplan-Meier Curve. *Journal of the American Statistical Association* 83, 414–425. doi: 10.1080/01621459.1988.10478612.

Kosovicheva, A., Ferreira, A., Vera-Diaz, F. A., and Bex, P. J. (2019). Effects of temporal frequency on binocular deficits in amblyopia. *Vision Res* 163, 52–62. doi: 10.1016/j.visres.2019.08.004.

Kwon, M., Wiecek, E., Dakin, S. C., and Bex, P. J. (2015). Spatial-frequency dependent binocular imbalance in amblyopia. *Sci Rep* 5, 17181. doi: 10.1038/srep17181.

Mao, Y., Min, S. H., Chen, S., Gong, L., Chen, H., Hess, R. F., et al. (2020). Binocular Imbalance in Amblyopia Depends on Spatial Frequency in Binocular Combination. *Invest Ophthalmol Vis Sci* 61, 7. doi: 10.1167/iovs.61.8.7.

Min, S. H., Mao, Y., Chen, S., He, Z., Hess, R. F., and Zhou, J. (2022). A clinically convenient test to measure binocular balance across spatial frequency in amblyopia. *iScience* 25, 103652. doi: 10.1016/j.isci.2021.103652.

South, J., Gao, T., Collins, A., Turuwhenua, J., Robertson, K., and Black, J. (2019). Aniseikonia and anisometropia: implications for suppression and amblyopia. *Clin Exp Optom* 102, 556–565. doi: 10.1111/cxo.12881.

Vera-Diaz, F. A., Bex, P. J., Ferreira, A., and Kosovicheva, A. (2018). Binocular temporal visual processing in myopia. *J Vis* 18, 17. doi: 10.1167/18.11.17.

Winn, B., Ackerley, R. G., Brown, C. A., Murray, F. K., Prais, J., and St John, M. F. (1988). Reduced aniseikonia in axial anisometropia with contact lens correction. *Ophthalmic Physiol Opt* 8, 341–344.
